# Overview of healthcare system in the Czech Republic

**DOI:** 10.1007/s13167-012-0139-9

**Published:** 2012-02-28

**Authors:** Judita Kinkorová, Ondřej Topolčan

**Affiliations:** 1 Technology Centre of the Academy of Sciences of the Czech Republic (Technology Centre ASCR), Ve Struhách 27, Praha 6, 160 00 Czech Republic; 2 Charles University Prague, E. Beneše 13, Plzeš 305 99 Czech Republic

**Keywords:** Czech Republic, Healthcare system, Politics, Financing, Predictive, Preventive and Personalised Medicine, Recommendations

## Abstract

The healthcare system in the Czech Republic underwent and still is undergoing dramatic changes since the Velvet revolution in 1989. History of the Czech healthcare system, main healthcare laws, and the current status of healthcare documented in the main healthcare indicators is described based on the several main sources as well as delivery of health services and the role of the main actors in healthcare system. The material is based mainly on Czech Health Statistics 2009, and HiT Summary, Health Care Systems in Translation, 2005, public information of Ministry of Health CR.

## Introduction

### Country description

**Figure 1:**
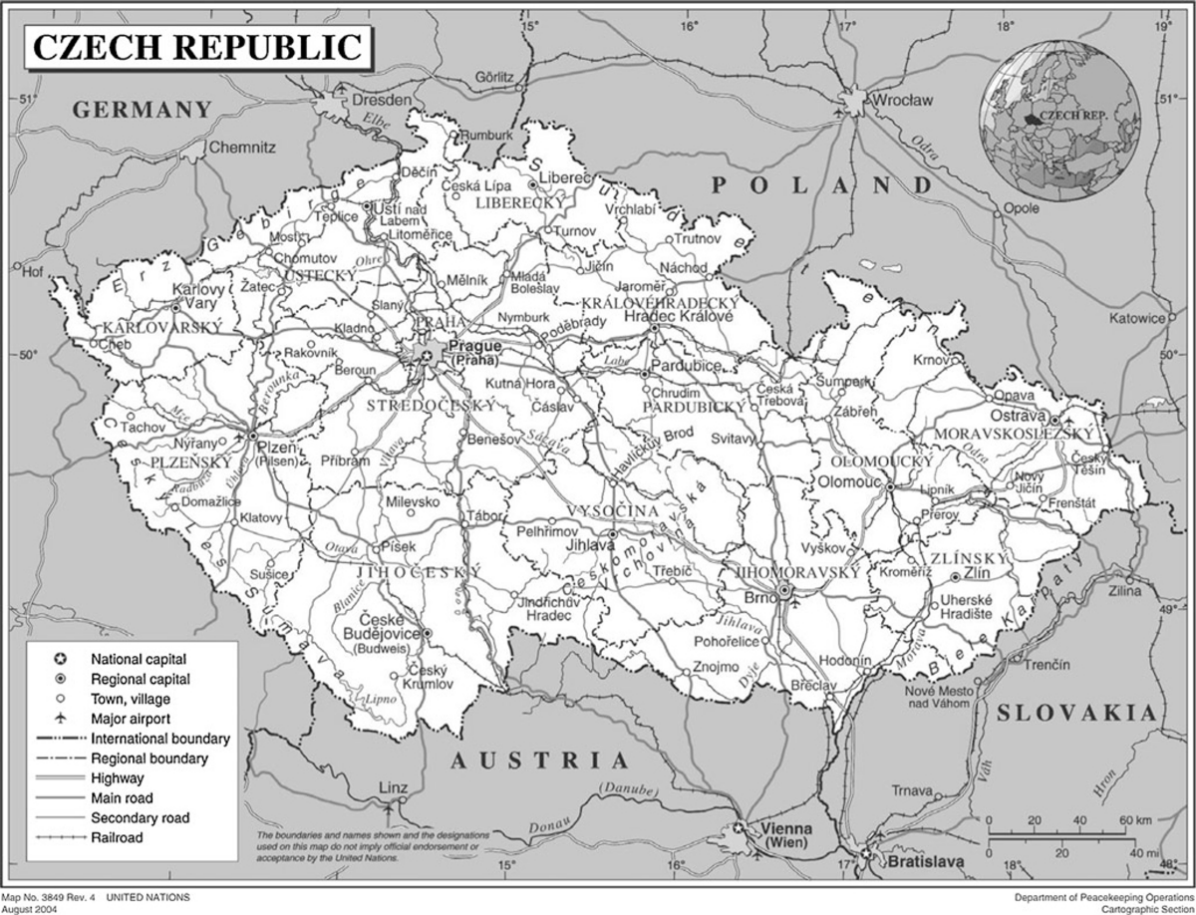
**Location of the Czech Republic in the Europe**. Taken from UN cartographic section.

The Czech Republic is centrally located in the heart of Europe, with the area of 78.865 square kilometres [1], and with an estimated population of 10,542,080 in 2011 (Figure 1), ethnically and linguistically Czech (94%). Other ethnic groups include Germans, Roma, Vietnamese, and Poles. Czech Republic has a democratic parliamentary system of government and a well-developed economy. The Czech Republic has been a member of the Organisation for Economic Co-operation and Development (OECD) since December 1995, a member of the North Atlantic Treaty Organisation (NATO) since February 1999 and a member of the European Union (EU) since May 2004 [1].

### History, government and political system [2]

After World War II, the political system in Czechoslovakia was greatly affected by the introduction of a Soviet-style Communist regime, as it was in the other countries of central and east Europe. In February 1948, the Communist Party became the only autonomous political entity.

After the revolutionary events, so-called "Velvet revolution" of November 1989 which brought about the downfall of the Communist regime, the entire country faced the uneasy task of resuming its pre-Communist traditions and building a democratic political system. A wide diversity of political parties was established even before the break-up of Czechoslovakia on December 31, 1992. The constitution of the Czech Republic, which became valid on the day of the birth of the new state, explicitly defined civil rights, the relationship between the executive and legislative branches of power, and the independence of the judiciary.

### Population and demography

With an estimated population of 10,542,080 at 30 June 2011, compared to 9.3 million at the beginning of the twentieth century, the population growth of the Czech Republic was limited and characterised by low fertility rates and loss of population in and around World War I and World War II. Population growth resumed until 1994 when the population was 10.3 million. From 1994 to 2005 natural growth was negative and the population decreased to 10.2 million. Since 2006, natural growth has been positive, but the most important factor for the recent population of the Czech Republic has been immigration, approximately 300,000 during the last decade [1].

**Figure 2:**
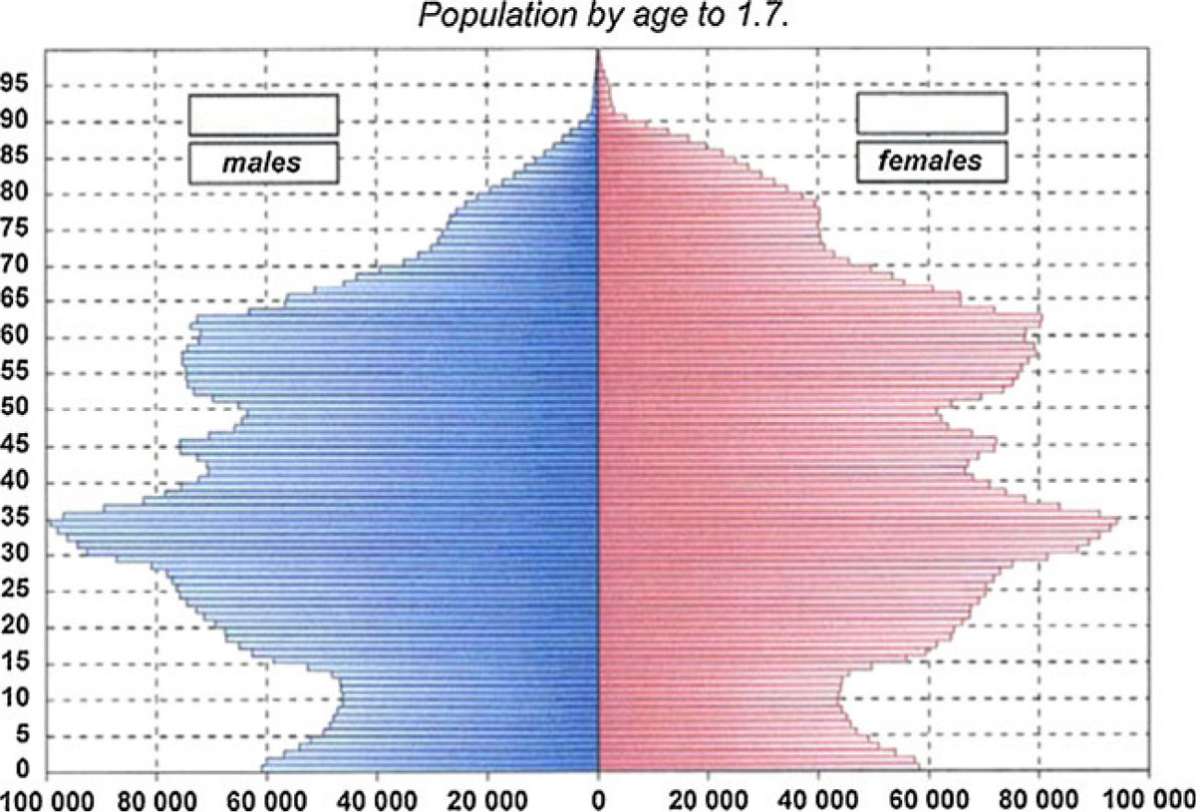
**Population by age to 1. July 2009**. Taken from Czech health statistics 2009 [1].

In 1994 the number of deaths exceeded the number of births for the first time since 1918, and the population is continuing to decline. Population by age in 2009 is shown in Figure 2.

Life expectancy at birth in the Czech Republic is continuously increasing, having reached 77.2 in 2009 [3].

The most frequent cause of death was and still is circulatory system disease, followed by malignant neoplasms. Despite the decrease of external causes (injury or poisoning) these as a group are the third largest cause of death in 2009. The forth most common cause of death are respiratory system diseases, followed by diseases of digestive system, diabetes mellitus, diseases of the blood and of the blood forming organs, tuberculosis and infectious and parasitic diseases.

Vaccination coverage in the Czech Republic is very high, greater than 97% in all relevant immunisation categories [4]. Vaccination against tuberculosis, diphtheria, tetanus, poliomyelitis and others are part of the compulsory childhood vaccination schedule.

The evolution of the Czech population (in 2009) is characterised by: the number of live births decreased, the number of deaths increased, the natural population increase remained positive, but was lower than in the previous year. Migration increase, about 28 thousand persons in 2009. The total population of the Czech Republic increased but its age structure grew older [5].

## History of healthcare system

History of healthcare system in Bohemia as a part of Austro-Hungarian Monarchy has the roots in 1883, when Otto von Bismarck established the health insurance as a part of the Bismarck's model of social security and health insurance system. After Czechoslovakia's independence in 1918, the Bismarck's healthcare system inherited from the Monarchy was expanded and well defined. After the World War II, Czechoslovakia becomes a part of Soviet empire of influence which had important negative impact on the healthcare system. In 1952 a soviet style centralised system of unified healthcare system, so-called Semashko model was introduced. The new system effectively solved the post-war problems of the early 1950s. In 1960s the modified Semashko's healthcare system reached a turning point and as a centralised and rigid in many aspects, it proved unable to respond flexibly to new health problems arising from life style changes and environmental factors [5].

The new era of healthcare system in Czechoslovakia started after so-called "Velvet revolution" in 1989. Czechoslovakia like other former soviet sphere countries opted for the introduction of a Bismarckian type of financing system which served as a transitional bridge toward more market-oriented mechanisms and institutions [6]. In 1990 and 1991, during the democratisation process, a dramatic liberalisation of the healthcare system took place. The principle of free choice of healthcare facility was introduced. In 1991, new laws were approved especially the General Health Insurance Act (No. 550/1991 Coll.), and the Act on the General Health Insurance Fund (No. 551/1991 Coll.). Since then, the healthcare system has moved towards a compulsory health insurance model, with a number of insurers financing healthcare providers on the basis of contracts.

From the early 1990s, considerable changes have been implemented in the Czech healthcare system. The majority of the planned changes have taken place and the implementation process has been remarkably smooth. A complete reconstruction of the healthcare facilities and authorities has been achieved and a health insurance system has been created. At the same time, there was an almost complete privatisation of primary healthcare, the pharmaceutical industry, pharmacies, healthcare support firms, spa facilities, etc. [7].

The healthcare system underwent a number of important changes since 2005: five of them are listed below

1. the implementation between 2005 and 2006 of a new risk adjustment scheme for redistributing social health insurance contributions among the health insurance funds;

2. the introduction in 2008 of user fees for doctor visits, hospital stays, prescription pharmaceuticals e.g., and out-of-pocket payments;

3. the inclusion in 2008 of the State Institute for Drug Control (Státní ústav pro kontrolu lečiv, SÚKL) in the process of setting maximum prices for pharmaceuticals for transparency of price settings;

4. the introduction in 2008 of a programme to supply accredited providers with additional financial support for training nurses and physicians;

5. an initiative to improve the quality of highly specialised care by identifying high performing healthcare facilities and allowing for special contractual conditions between these facilities and the health insurance funds.

## Organisation of health system in the Czech Republic

Czech health system is based on five pillars:

1. solidarity

- solidarity between healthy people and the sick is fostered in healthcare systems by separation between the provision of healthcare and its financing

- solidarity of the economically active with the economically inactive people means that every insured person pays an insurance premium as a percentage of their income regardless of what healthcare they receive or will receive;

2. high degree of self-administration;

3. multisource financing with major share of public health insurance. Healthcare is funded from public health insurance, direct payments, the national budget and regional budgets;

4. equal availability of healthcare for all insured persons. The healthcare system strives to create conditions in which there are no differences in the availability of healthcare;

5. obligatory vaccination against infectious diseases.

These pillars are legally supported by following selected laws:

- Resolution of the Presidium of the Czech National Council 2/1993 promulgating the Charter of Fundamental Rights and Freedoms as part of the Czech constitutional order

- Act 48/1997 Coll., on public health insurance, amending some related laws

- Act 20/1966 Coll., on public healthcare, amending some related laws

- Act 592/1992 Coll., on premiums for general health insurance, amending some related laws

- Act no. 258/2000 Coll., on public health protection and amendments to several related acts, as amended [8].

Healthcare is in the Czech Republic is provided by structured network of several types of healthcare, some of them are listed below [6,8]:

### Outpatient care

Outpatient care is provided by primary care physicians or specialists. If a person is taken ill, they usually contact a primary care physician working near their home. These are general practitioners for adults, general practitioners for children and young people, dentists and gynaecologists.

When choosing a physician, you should bear in mind that you can only register with a physician who has concluded a contract with your insurance company.

If healthcare is to be reimbursed from public health insurance, the insured must first register with a primary care physician (the local basic healthcare provider). A physician may only refuse to register an insured person if the registration brought to the physician so much work that it would not permit provision of quality care to the patient or other patients in the physician's care.

A patient can visit a specialist physician in the Czech Republic without a referral from the primary care physician.

### Institutional (inpatient) care

If the nature of an illness demands this, a primary care physician or outpatient specialist can refer a patient for hospital treatment or arrange for their admittance.

Inpatient care is provided in hospitals and specialised institutions, such as psychiatric hospitals and rehabilitation centres, hospices, sanatoria, long-term care hospitals.

### Ambulance and emergency rescue service

Emergency rescue service is available to deal with cases of acute illness or accident when a patient cannot get to a physician and immediate treatment is needed and transport of the patient to a healthcare facility under permanent care to prevent further aggravation of their health conditions or threat to the life.

### Balneological care

Balneological care can be regarded as an essential part of the curative process. It is recommended by one's attending physician and confirmed by a reviewing physician. Entitlement to balneological care is claimed on a pre-printed form by the registering general physician or attending physician in case of hospitalisation.

### Long-term care

Long-term care for older or disabled people is provided in two overlapping settings with different systems of organisation and funding: residential long-term care facilities and other social services financed from central, regional or municipal budgets, and healthcare facilities for long-term inpatient care financed primarily through the social health insurance.

### Mental healthcare

Mental healthcare is provided both in the ambulatory settings and in inpatient facilities which include hospital psychiatric departments, psychiatric hospitals and psychiatric institutes.

### Dispensing medicaments and medical devices

In the Czech Republic there is an extensive network of pharmacies dispensing medicaments and medical devices, both on prescription and over the counter.

### Preventive care

Preventive examinations and vaccination against infectious diseases are performed by primary care physicians.

## Financing

**Figure 3:**
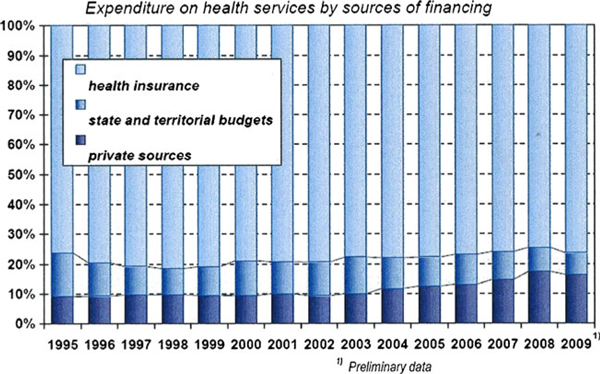
**Expenditure on health services by sources of financing**. Taken from Czech health statistics 2009 [1].

In 2010, as in the preceding years, the predominant part of health expenditure was financed by the public health insurance system covering 76.6% of the total (in Figure 3 is shown expenditure on health services by sources of financing). The State and territorial budgets covered 7.2% and private expenditure covered 16.2%. The share of private expenditure in the total expenditure on health rose particularly after 2008, due to new regulation fees in health services. In 2010 the private expenditure on health slightly decreased, partly as a result of softening of the regulation fees and of stagnant purchasing power of the population. The total expenditure on health increased from 2009 by 821 million CZK and in 2010 it amounted in absolute value to 290 412 million CZK, i.e. 27 613 CZK per 1 inhabitant. This total expenditure represents 7.69% of the GDP in 2010. Public expenditure, i.e., that of the public budgets and of the public health insurance system, totally 243 283 million CZK, by 1 646 million CZK more than in the preceding year (all data are preliminary) [9].

Healthcare in the Czech Republic is provided primarily on the basis of statutory health insurance, which is currently provided by nine health insurance funds.

The largest health insurance fund - the General Health Insurance Fund, GHIF - has 77 district branches. Any person with a permanent residence in the Czech Republic is entitled to health insurance.

Healthcare services are covered by the health insurance funds. The following services are fully or partially covered by health insurance [8]:

1. outpatient and institutional (inpatient) care,

2. emergency and ambulance services,

3. preventive care,

4. dispensary care,

5. supply of medicaments, medical supplies (e.g. hearing aids, bandages),

6. balneological care, care in specialised children's hospitals and sanatoria,

7. industrial healthcare,

8. transport of the sick, reimbursement of travel expenses,

9. deceased examination and autopsy.

There are many procedures which insured persons co-finance. These are procedures or medical devices provided outside the legal framework. Some cases in point are dental procedures, some balneological care and some medicaments. Some medicaments are reimbursed in full by insurance companies whereas some are co-financed by the patients. In every category of medicaments there must be at least one reimbursed in full by an insurer. Costs of medicaments and medical devices during hospitalisation are reimbursed in full by the insurer and the insured person does not pay directly.

## International comparison

**Figure 4:**
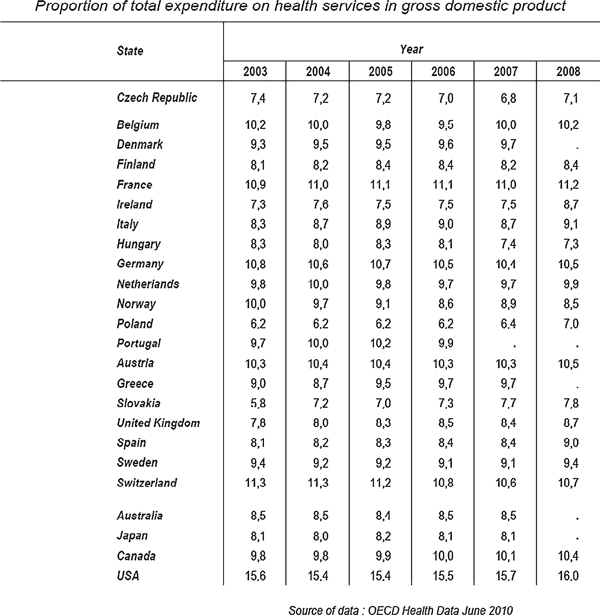
**Proportion of total expenditure on health services in gross domestic product (GDP)**. Taken from Czech health statistics 2009 [1].

This part contains comparison of the shares of expenditure on health in gross domestic product (GDP) in selected countries in Europe (Figure 4). The source of these data is the Database OECD Health Data 2011.

Ageing of the population is a phenomenon that characterises in the long term the demographic evolution of most European countries. As a result of the decreasing natality the share of persons older than 64 years increases.

The age preference index, defined as the number of persons aged 65 years or more per 100 children, exceeds the value 100 in increasing numbers of countries. It means that there are more seniors than children. This is already so in 3/5 of EU member countries, most markedly in Germany and in Italy. On the opposite pole are the "youngest" countries, Albania, Ireland and Iceland, where the share of seniors is only about one half of its value in Germany and Italy.

The Czech Republic belongs to countries with a low share of children up to 15 years old (14.2% in 2009). With its share of persons in the age group of 65 or more years (15.0% in 2009) the CR still does not reach the European Union average that exceeded 17% in 2007. In 2006 the Czech Republic joined the group of countries with more persons over 64 than children. The age preference index in 2009 reached the value 107.

Another common feature of most European countries is the decreasing mortality connected with growing length of human life. The average value of standardised death rate (that eliminates the influence of the age structure of the population; further SDR) shows a long-term decreasing trend in European Union (EU) as well as in EU-15 (member countries before May 2004) and in EU-12 (new member countries that joined EU in 2004 and 2007). The average values of the total SDR in EU-12 exceed the averages in EU-15 by 2/3 in men and by almost 1/2 in women. The SDR values in the Czech Republic, in spite of the marked decrease in the past two decreases, still exceed not only the averages of EU-15 (by almost 40% in men and by ca. 30% in women), but also of the whole EU, by 1/5 both in men and women.

## Current status of the PPPM in the Czech Republic

There is no conception of PPPM (predictive, preventive and personalised medicine) in the Czech Republic officially proposed by policy makers as e.g. Ministry of Health of the Czech Republic (MoH), and National Institute of Public Health (SZÚ), Ministry of Labour and Social Affairs (MoLSA), and other governmental bodies. The main document regarding the national health policy is "Ministry of Health Departmental Program for Research and Development III (DPR III)" [10] with some aspects regarding basic principles of PPPM. As an example of this approach the SZÚ is responsible for preventive services covering:

1. compulsory vaccination and preventive examinations for children of specific age groups

2. compulsory vaccination and periodic examinations by general practitioner very two years

3. well organised cancer screening programmes (e.g. colo-rectal cancer, breast cancer, cervical cancer)

4. mammography for women 45-69 every two years, preventive gynaecological examinations including cytology from the age of 15

5. regular vaccination covering 99% of the population (tuberculosis, tetanus, diphtheria, pertussis, poliomyelitis, measles, mumps, and rubella, cervical cancer, from 2001 also hepatitis B, and *Haemophilus influenzae *type B [5].

Several additional preventive care services are delivered to the Czech citizens: reducing alcohol harm, treating drug addiction, tackling obesity especially in children, prevention of smoking, sexual health education, life style health risks.

The current situation in the Czech Republic is characterised by raising awareness and recognition of PPPM, promotion of education and up to date information.

Education of PPPM is one of the initial activities. The only educational centres are universities and research institutes. The concept of PPPM national education is still under preparation at both non-professional (general) and professionals in personalised medicine.

Following steps will include graduate, post graduate and continuing education programmes for young and experienced researchers and medical staff those who will be taking the PPPM forward in the coming years.

## Recommendations

Basic principles of PPPM should be incorporated into basic strategic documents of policy makers (MoH, SZÚ, MoLSA), with ethical, legal and social issues involved.

Educational programmes for students and professionals at all levels in conventional and molecular diagnostics, biomedicine, biotechnologies, ethics, and economics, for universities, research units, private and public hospitals, and public--patients and their family members with all necessary materials should be systematically prepared.

All relevant partners like policy makers, stakeholders, pharmacy, biomedical industry, universities, research centres, hospitals, patient, and patients' organisations should be involved in process of establishing PPPM in the Czech Republic.

Czech professionals should be more involved in international PPPM programmes and research and other activities, e.g. Framework Programme 7 (FP7) and be more active in preparation of the following programme HORIZON 2020.

Czech professionals should more participate at all international activities like congresses, conferences, workshops, Joint programming, be more active in publishing.

## Conclusions

Since 1989 the Czech healthcare system has undergo major and important changes with the aim to start and accelerate process of democratisation and humanisation of the healthcare system and make it more efficient. The next important step was to separate financing of healthcare from the state budget.

The key challenge to health reform in the upcoming period is to improve high quality of healthcare to all inhabitants of the Czech Republic.

The process of transformation is on-going process well-coordinated with the basic principles of EU strategy in healthcare in the member states.
